# The *Bhagavad Gita*, Psychological Safety, and the Medical Learning Environment: Hitting the Sweet Spot

**DOI:** 10.1016/j.jacadv.2024.101458

**Published:** 2024-12-11

**Authors:** Anuradha Lala, Logan D. Cho, Ankur Kalra

**Affiliations:** a Mount Sinai Fuster Heart Hospital, Department of Population Health Science, Icahn School of Medicine at Mount Sinai, New York, New York, USA; b Department of Medicine, Columbia University Irving Medical Center, NewYork-Presbyterian Hospital; c Franciscan Health, Lafayette, Indiana, USA; d Krannert Cardiovascular Research Center, Indiana University School of Medicine, Indianapolis, Indiana, USA

**Keywords:** learning, medicine, teacher, *Bhagavad Gita*, psychological safety

## Abstract

The art of medical education is increasingly emphasized in academic medicine as we have begun to appreciate how much potential there is for improvement from traditional paradigms. The medical learning environment poses unique challenges wherein the transmission of information from teacher to student is critical and yet psychological safety is a core principle required for effective learning. Drawing from the *Bhagavad Gita*, this piece seeks to highlight how Lord Krishna, the teacher, and Arjuna, the learner, depict the idyllic teacher–student relationship, exemplifying the essence of psychological safety. This article offers several practices clinical educators can imbibe to maximize psychological safety in the medical learning environment at distinct phases of learning: prelearning, learning, and postlearning. Ultimately, effective learning is a bidirectional process that can be a fulfilling and rewarding experience for the student and teacher alike, where all spirits are uplifted.

## Corresponding Author

**Figure:**
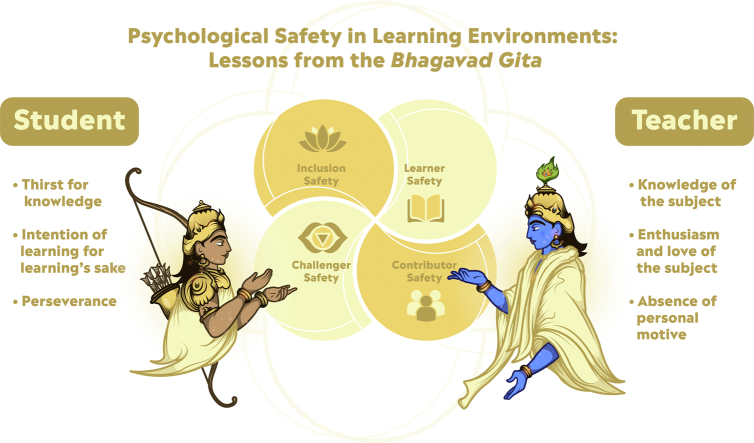

May Divine energy protect us both together; may It nourish us both together;

May we work conjointly with great energy,

May our study be vigorous and effective;

May there be no ill will between us;

Om peace peace peace.

- Ancient Vedic mantra, Upanishads

(∗often referred to as the teacher/student mantra)

## The Opportunity

The human body is a miracle.

As physicians, we are gifted with the opportunity to learn about its myriad functions, to protect it, and to care for it. In this process, the learning is both vast and eternal—noble in purpose, intimidating in scope. Books provide necessary information, but we rely on clinician educators to teach medicine by example. We observe, absorb, practice, and mature in the practical application of medical knowledge at the bedside. There is no formula, no one “right way” to teach, or to learn medicine. This lifelong process renders vulnerability, heartache, and exhilaration—necessitating a rigorous and safe system to optimize both learning and patient care.

Along the way, we come across mentors and clinical educators whose styles we find personally effective, and whose interactions with patients leave us with durable lessons that stay with us for years. These teachers seamlessly integrate medical knowledge and patient management in an inimitable manner, weaving complex biomedical concepts with extraordinary care and compassion. Such experiences can be uniquely gratifying, and almost intimate, as we all develop our own skills and styles.

We all also experience educational scenarios that may feel counterproductive to effective learning. Such educators or circumstances may trigger excessive anxiety or pressure, thereby creating a suboptimal learning environment, and potentially giving rise to negative emotions of apathy, frustration, or even dejection. So how then, do we optimize the bidirectional educational experience for both students and teachers in the modern-day field of medicine?

## Psychological Safety in the *Bhagavad Gita*

One key principle that might offer a way forward is that of psychological safety. Psychological safety is the belief that one can bring up ideas, questions, or doubts without a fear of negative repercussions. This concept has been organized into 4 elements that sequentially build on one another. The first is *Inclusion Safety*—where one feels valued and part of the team regardless of position. The second is *Learner Safety*—where one is comfortable asking questions, experimenting, and learning from mistakes. The third is *Contributor Safety*—where one feels invited and expected to work as a key team player. The fourth is *Challenger Safety*—where one feels comfortable contesting the status quo, to express ideas, and raise problems. The delineation of these levels of safety allows for enhanced emotional awareness and regulation in optimal interpersonal exchanges.^1^

**Figure 1:**
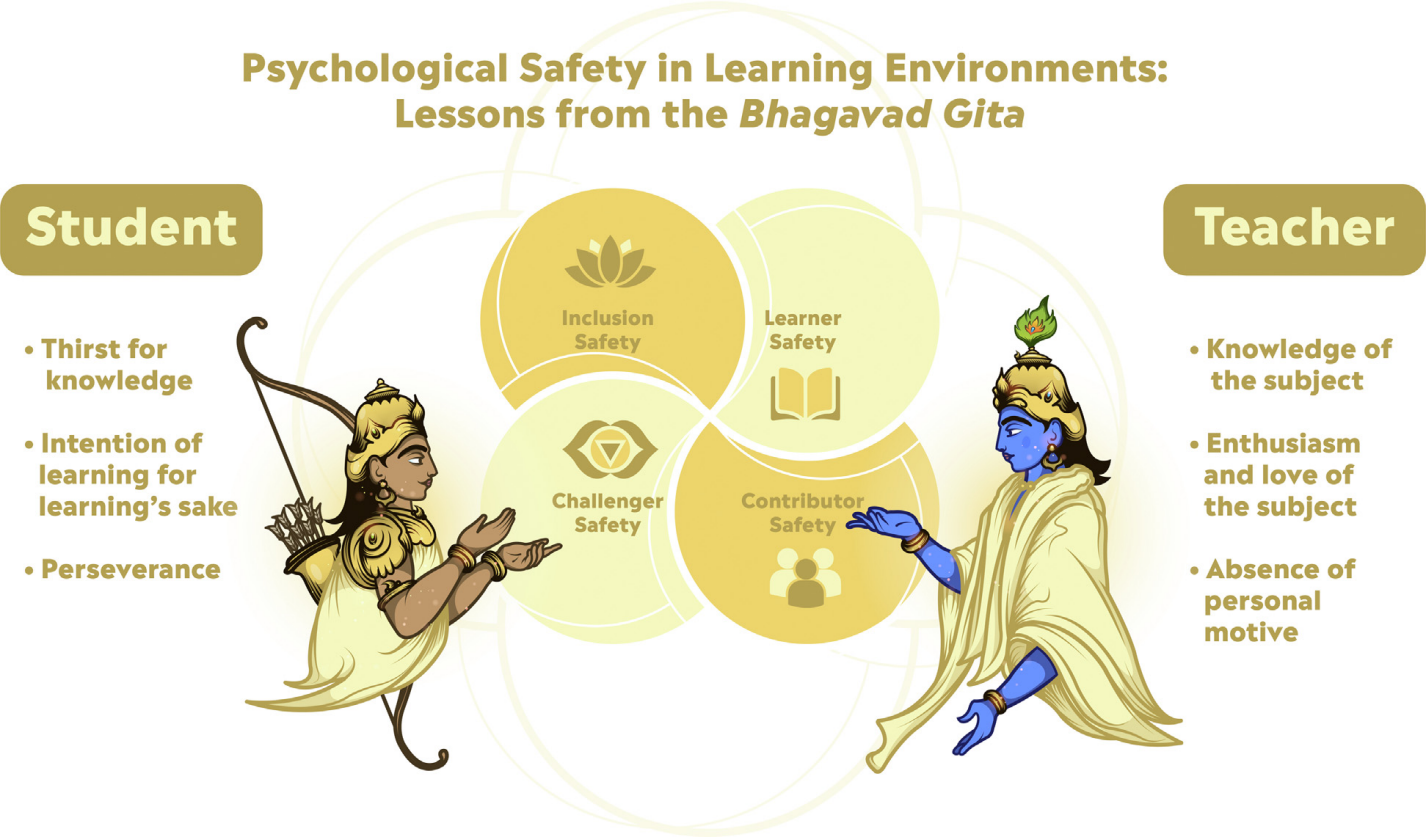
**Psychological Safety Amid Lord *Krishna*, the Divine Teacher and Charioteer, on the Battlefield of *Kurukshetra* With *Arjuna*, the Warrior and Student** Arjuna as the learner demonstrates deep reverence for the teacher, Lord Krishna. Yet, Arjuna finds the freedom to ask questions rising from curiosity and doubt. Lord Krishna treats Arjuna as an equal, encouraging debate and dialogue, and fostering deep-rooted learning through self-inquiry—rooting the learning exchange in psychological safety.

The *Bhagavad Gita* provides key insights to the ultimate and ideal learning exchange between the teacher (Sri Krishna, the celestial Lord) and the learner (Arjuna, the warrior, seeker, devotee, and friend) on the battlefield. Here, Arjuna seeks refuge in Krishna, wanting to retreat from fighting his kin on the opposing side. Lord Krishna proceeds to relay the essential philosophies of life—performing actions for the actions’ sake, remaining detached from the results, and aligning oneself with the flow of the universe through awareness in acceptance. Such concepts are challenging to grasp and accept, let alone put into practice. How then did this divine educational interchange occur? Here we see each element of psychological safety exemplified (Figure 1). Throughout the discourse of the *Bhagavad Gita*, Lord Krishna never scolds, rebukes, or diminishes Arjuna. He only uplifts him and reminds him of his true and divine nature. The Truth the teacher Krishna bestows is overwhelming—and beyond the mental, physical, and emotional realm. Yet, Arjuna feels safe to ask questions (*Learner/Inclusion* safety), participate in dialogue (*Contributor* safety) and even challenge (*Challenger* safety) his teacher to better understand His supreme teachings. Lord Krishna holds His responsibility in teaching sacred. He answers questions methodically and thoroughly, adjusting his words and examples to meet Arjuna’s depth perception.

Interestingly, the great philosopher Swami Vivekananda described the essential attributes and intentions of both the student and teacher (demonstrated in the *Bhagavad Gita*) that give way to an ideal learning exchange rooted in safety^2^: Arjuna, who represents the ideal student, has 1) a thirst for knowledge; 2) a genuine intention to learn; and 3) the perseverance to overcome obstacles. These are the attributes that we as lifelong students should aspire to embody. Similarly, Lord Krishna represents the ideal supreme teacher has 1) a deep knowledge of the subject matter; 2) an innate enthusiasm and love of the subject; and 3) the absence of personal objective. He is teaching without a hidden, ulterior objective. These are the attributes that we must petition of ourselves as lifelong educators. Even if seemingly impossible, the mere intention of these guideposts may allow for a higher likelihood of psychological safety in the learning environment, and thereby a potentially higher likelihood of hitting the “sweet spot”.

## Challenges and Practical Considerations

Medical students and trainees are a largely self-selected population of exceptional learners. Still various pressures, personalities, and motivating factors contribute to variability in educational experiences. The value of learning about advanced heart failure management may vastly differ between an ophthalmology-bound medical student on a required clerkship and a cardiology-bound internal medicine resident on a chosen elective block. Understanding differing motivations with tailored teaching goals and expectations facilitate mutual harmony.

The learner's perception of the teacher also has significant implications. Cultivating an environment that strikes a balance between respect and permission is a difficult but vital charge. Too much respect with too little permission yields paternalism, whereas too much permission with too little respect results in exploitation. Too little of both respect and permission prohibits learning altogether. Arjuna, the student, had deep reverence for Lord Krishna. However, he also felt comfortable asking questions in response to explanations of concepts. Lord Krishna's only motivation was the true enlightenment of his student, Arjuna. In this exchange, Lord Krishna treated Arjuna as his colleague, inviting debate and doubt, while also encouraging Arjuna to explore enlightenment through self-inquiry.

The other balance to be sought is that between psychological safety and accountability. Accountability breeds motivation and potentially healthy stress. Excessive safety without accountability fosters complacency without growth; excessive accountability without adequate safety cultivates anxiety, fear, and unhealthy stress. Amid the many clinical and academic demands upon our time and energy, what are the practical ways that we can foster this kind of culture as educators? Here, we offer several practical steps for consideration in the context of 3 phases: prelearning, learning, and postlearning.

The prelearning phase involves introductions, articulating intentions and goals, and establishing core values. Alarms, messages, time constraints of rounds, and frequently changing teams all characterize the unique-to-medicine cognitive load that often complicates opportunities for well-organized introductions. To this effect, dedicating time to conduct a formal needs assessment of the learner—verbal or written—offers an early invitation for the learner to share their knowledge level and background. In this phase, it is helpful to agree on and articulate team expectations, intentions, and goals for the learning period. Having a rehearsed checklist of expected team values can streamline introductions amid chaotic atmospheres. Participation and curiosity should be explicitly encouraged with an emphasis on the intention to prioritize safety and respect as core values of the team, which aids in building trust. Overall, this initial interaction should make the learner feel informed, included, and expected to engage as a valued team member.

Once introductions have been made, core values have been established, and knowledge levels have been assessed, the learning phase commences. The learning phase comprises the majority of the experience together and can range in duration from a few hours to several weeks. It is in this phase of learning where we can strive to imbibe the attributes of Lord Krishna. Here, a passionate teacher with deep knowledge of the subject material can aim to impart knowledge on the learner. As shared previously, Lord Krishna was free of ulterior motive and was purely interested in the learning of his student Arjuna. Active effort is made to include, solicit participation, and even encourage a challenging of what is taught. It is important to acknowledge that consistent implementation can be a struggle, especially in the medical environment, as competing goals and obstacles are continually encountered. Many times educators may need to cultivate distinct opportunities in which the students’ learning is the priority, without sacrificing the care of patients that we seek to honor and uphold to the highest standards.

The postlearning phase, consisting of feedback and assessment at the completion of the learning period, is the capstone of the learning experience. This is another area where external factors can complicate execution, but time should be dedicated for reflection and formal closure of the experience. In addition to an exchange as to competency, performance, and areas for improvement, there should also be a review as to what levels of psychological safety were achieved and whether a culture of respect and belonging was upheld. Bidirectional communication is imperative for the growth of both the student and the teacher, outlining opportunities for improvement for subsequent experiences. When multiplied across a series of learners, this process offers the teacher a substantive sample to evaluate, opening the door for self-iteration as warranted. Though we increasingly find ourselves in the roles of educators as we age, it is our Hippocratic creed to be eternal learners in medicine, including challenging ourselves to become more effective educators.

With the outlined “go-to” steps in mind, we can collectively foster psychological safety—both systematically and consistently, with intentionality and humility to uplift students and teachers alike. In so doing, the culture of learning in medicine can meet its full potential: to support exchange and practice of knowledge for the health of our patients and for the elevation of our spirits in service.

## Funding support and author disclosures

The authors have reported that they have no relationships relevant to the contents of this paper to disclose. They also humbly submit the thoughts expressed in this piece are an offering based on their personal reflections, and are not direct translations from the *Bhagavad Gita*.
